# Using implementation facilitation to foster clinical practice quality and adherence to evidence in challenged settings: a qualitative study

**DOI:** 10.1186/s12913-017-2217-0

**Published:** 2017-04-20

**Authors:** Mona J. Ritchie, Louise E. Parker, Carrie N. Edlund, JoAnn E. Kirchner

**Affiliations:** 1 Department of Veterans Affairs, VA Quality Enhancement Research Initiative (QUERI) Program for Team-Based Behavioral Health, 2200 Ft Roots Dr, Bdg 58, North Little Rock, AR 72114 USA; 2 Department of Psychiatry, University of Arkansas for Medical Sciences, 4301 W Markham St, #755, Little Rock, AR 72205 USA; 3 Department of Management and Marketing, College of Management, University of Massachusetts, 100 Morrissey Blvd, Boston, MA 02125 USA

**Keywords:** Facilitation, Fidelity, Quality, Expert ratings, Implementation science, Primary care, Mental health, Integrated care

## Abstract

**Background:**

We evaluated a facilitation strategy to help clinical sites likely to experience challenges implement evidence-based Primary Care-Mental Health Integration (PC-MHI) care models within the context of a Department of Veterans Affairs (VA) initiative. This article describes our assessment of whether implementation facilitation (IF) can foster development of high quality PC-MHI programs that adhere to evidence, are sustainable and likely to improve clinical practices and outcomes.

**Methods:**

Utilizing a matched pair design, we conducted a qualitative descriptive evaluation of the IF strategy in sixteen VA primary care clinics. To assess program quality and adherence to evidence, we conducted one-hour structured telephone interviews, at two time points, with clinicians and leaders who knew the most about the clinics’ programs. We then created structured summaries of the interviews that VA national PC-MHI experts utilized to rate the programs on four dimensions (overall quality, adherence to evidence, sustainability and level of improvement).

**Results:**

At first assessment, seven of eight IF sites and four of eight comparison sites had implemented a PC-MHI program. Our qualitative assessment suggested that experts rated IF sites’ programs higher than comparison sites’ programs with one exception. At final assessment, all eight IF but only five comparison sites had implemented a PC-MHI program. Again, experts rated IF sites’ programs higher than their matched comparison sites with one exception. Over time, all ratings improved in five of seven IF sites and two of three comparison sites.

**Conclusions:**

Implementing complex evidence-based programs, particularly in settings that lack infrastructure, resources and support for such efforts, is challenging. Findings suggest that a blend of external expert and internal regional facilitation strategies that implementation scientists have developed and tested can improve PC-MHI program uptake, quality and adherence to evidence in primary care clinics with these challenges. However, not all sites showed these improvements. To be successful, facilitators likely need at least a moderate level of leaders’ support, including provision of basic resources. Additionally, we found that IF and strength of leadership structure may have a synergistic effect on ability to implement higher quality and evidence-based programs.

**Electronic supplementary material:**

The online version of this article (doi:10.1186/s12913-017-2217-0) contains supplementary material, which is available to authorized users.

## Background

In this article, we report our evaluation of a facilitation strategy to help clinical sites implement evidence-based practices in the context of the Veterans Health Administration (VHA) Primary Care-Mental Health Integration (PC-MHI) initiative. Implementation of evidence-based practices and programs (EBPPs) within healthcare systems can be widely variable. Numerous theories and substantial research evidence indicate that organizations and the individuals within them are often slow to adopt new practices (e.g., [1–6]). Such variability can result in lack of fidelity to evidence, poor program quality, and, ultimately poor outcomes. To spread EBPPs system-wide, many organizations simply mandate global implementation [7]. Such mandates may increase organizations’ motivation to change but not affect their capacity for change [8]. VHA, the largest integrated healthcare system in the United States, has been a forerunner in the development and promotion of EBPPs through clinical initiatives that include mandates, as well as other supports for change [9–11]. To transform mental health care for Veterans, VHA has provided mental health enhancement funds to increase staffing and has mandated provision of multiple specific EBPPs across VA healthcare facilities [12]. Sometimes mandates are poorly implemented because they receive little national implementation support [13, 14]. On the other hand, even when mandated EBPPs are highly supported, implementation can still be variable; this has been the case for several VHA programs including suicide prevention [11] and evidence-based psychotherapy treatments [9, 15]. VHA has also invested significant resources in its PC-MHI initiative [10]. Similar to the previous examples, all VA medical centers have implemented PC-MHI to some extent but not all have fully implemented evidence-based care models mandated by VA [10].

Variable implementation of EBPPs is likely inevitable given the dynamic and complex nature of healthcare systems and the vast number of factors that can foster or impede implementation success [16–18]. There is an extensive body of theory and evidence regarding contextual factors that affect organizations’ ability to change and implement new practices. For example, engaged and supportive leaders can sanction changes, reinforce priorities and authorize necessary resources for implementation efforts [19, 20]. An organizational climate that fosters stakeholder participation, innovation and collaboration can support EBPP adoption [8, 21]. Organizations need resources for staffing, education and training, and space; and they need the knowledge and capacity for planning change and monitoring and improving implementation processes; and stakeholders need protected time for implementation efforts [8, 22]. Stakeholder perceptions of the need for an innovation, the relative advantage of implementing it and the innovation’s fit with existing values and goals can also impact implementation efforts [16]. The presence of these contextual factors and many others are widely variable across healthcare organizations. Additionally, interactions between different contextual elements can vary as well [16, 23]. Although all healthcare settings experience implementation challenges, some may experience far more than others, lacking sufficient resources, infrastructure support and process knowledge needed to implement innovations [24].

Facilitation, a multi-faceted process of enabling and supporting individuals and groups, has been widely used in clinical settings, particularly primary care settings, as a strategy for overcoming barriers and leveraging strengths to foster implementation of EBPPs, prevention services and innovative care delivery models [24–26]. Facilitators can apply implementation science knowledge and interventions to help and enable others to understand what they need to change, plan and execute changes and address barriers to change efforts. Specifically, facilitators work with teams of key stakeholders to select innovations, adapt them to the local context, and select or develop interventions (e.g., formative evaluation, audit and feedback, clinical reminders) and tools (e.g., pocket cards, decision support) to support implementation [27–31]. Although facilitation has been widely used to address implementation challenges, we know little about whether this implementation strategy can help clinical settings with the most challenging contexts successfully implement PC-MHI programs. We applied and evaluated a facilitation strategy within the context of VHA’s PC-MHI initiative [10] to help sites with challenging clinical contexts implement PC-MHI and build capacity for future implementation efforts. This article describes one component of our evaluation of the implementation facilitation strategy, the assessment of the relative quality, fidelity to evidence, sustainability and likely level of improvement in PC-MHI programs.

### Study context

VHA’s mandate to implement PC-MHI required that all VA primary care clinics serving more than 5000 Veterans implement a blend of two complex, evidence-based PC-MHI care models [12]. Both models require significant changes in the structure and processes of mental health care delivery. In one of the models, co-located collaborative care, mental health providers, embedded in primary care clinics, offer brief assessment, diagnosis and treatment for Veterans with mental health and substance abuse care needs [32, 33]. In the other highly evidence-based model, care management, care managers assist primary care clinicians in the care of this population by utilizing guideline-based protocols to provide clinical assessment, patient education and activation and follow-up care [34–36]. VA’s mandate to implement PC-MHI recommends, but does not require, that facilities implement either Translating Initiatives for Depression into Effective Solutions (TIDES) or Behavioral Health Laboratory (BHL), two specific care management models that have proven efficacious and effective within VA populations [37, 38]. VA delegated responsibility for PC-MHI implementation to regional network leaders and established a national program office to provide consultation, technical support, education and training, identification and dissemination of best practices, and development and dissemination of tools to support PC-MHI program implementation [39].

This study was part of larger VA-funded project (HSR&D SDP 08-316, J, Kirchner, PI), where we applied and evaluated an implementation facilitation (IF) strategy both to implement and foster sustainment of PC-MHI programs at sites that would have been unable to implement PC-MHI without assistance. When the project began in 2009, many VA facilities had not implemented PC-MHI. We believed that some sites, in time, would be able to implement PC-MHI but those that lacked sufficient resources, infrastructure support and process knowledge would need help. The IF strategy incorporated a blend of external and internal facilitation [40]. A national expert (JEK) in implementation science, PC-MHI models and evidence for them, and facilitation strategies, worked in tandem with network-level internal regional facilitators to support PC-MHI implementation. Facilitation activities included multiple evidence-based implementation strategies. The frequency and timing of facilitators’ application of these strategies varied by site based on the phase of the implementation process, site-level contextual factors and the perspectives of local site stakeholders regarding the evidence supporting PC-MHI [41, 42]. We describe the IF strategy, including facilitation activities, in the methods section.

The project was comprised of three primary studies. In one, we demonstrated that compared to non-IF clinics, primary care patients at IF clinics were significantly more likely to be seen in PC-MHI, primary care providers were more likely to refer patients to PC-MHI, and a greater proportion of those providers’ patients were referred to PC-MHI [43]. In a second study, we conducted a qualitative analysis of how the facilitation intervention unfolds over time and in response to local context. We also explored how it fit into a broad conceptual framework for planned organizational change [41, 42, 44, 45]. In the third study, described in this article, we explored two questions: 1) whether IF could help clinics with challenging contexts to implement PC-MHI programs and 2) whether IF could foster development of PC-MHI programs that are of high quality, adhere to evidence, are sustainable and are likely to lead to improvement of clinical practices and outcomes as assessed by experts. Because of our small sample size we did not have sufficient power to conduct statistical analyses. Thus we employed and present a qualitative assessment of our findings.

## Methods

We applied qualitative descriptive methods to assess whether IF can foster implementation of PC-MHI programs, including facilitating the ability to create high quality programs that adhere to evidence. An independent evaluation team conducted all research activities and facilitators were blind to the evaluation findings until after the intervention ended. The VA Central Institutional Review Board approved the research, which was conducted between February 2009 and August 2013.

### Study setting

We conducted the study in sixteen VA primary care clinics located in four VA networks. We matched clinics at both the network and clinic levels, resulting in two matched pairs of networks and eight matched pairs of clinics (four clinics within each network). Because our IF strategy and site recruitment were complex and the intervention occurred within a rapidly unfolding policy context, we could not wait to select all four networks before condition assignment. We therefore assigned the first two networks to the IF intervention. We then matched these two networks to two others for comparison purposes. It was not important when the comparison networks entered the study because we allowed the natural process to unfold in these networks without interference. Given these factors, however, randomization was not possible. Because the structure and operational authority of network-level leadership can influence efforts to facilitate implementation of PC-MHI, we selected one IF network (A) and one comparison network (B) with strong mental health service line structures [46]. Mental health leaders in these networks had dedicated budgets and input into the selection and evaluation of VA Medical Center (VAMC) mental health leaders and network policies and procedures. In the other networks, IF network C and comparison network D, mental health leaders did not have a dedicated budget and had only very limited input into selection and evaluation of VAMC mental health leaders and network policies and procedures. Although all network mental health leaders were extremely supportive of PC-MHI implementation, we wanted to control for the influence of network mental health service line structure on implementation. Additional information about the network selection and matching process is described in a previous publication [43].

To select clinics in IF networks, mental health leaders, based on their knowledge and experience with implementation of PC-MHI and other clinical initiatives at facilities in their networks, identified clinics that 1) had not yet implemented and would have difficulty implementing a policy-compliant PC-MHI program without assistance, 2) delivered services or had the potential to deliver services to 5000 or more primary care patients a year, and 3) planned to implement a PC-MHI program in the first year of the study. To select clinics in comparison networks, mental health leaders identified a list of clinical sites using the same clinic selection criteria. We then selected clinics from that list that best matched IF clinics on facility type, clinic size, number of primary care providers, clinic location (rural/urban), academic affiliation, perceived need for PC-MHI and innovativeness [43]. In each network, one of the clinics was located at a VAMC and the other three were community based outpatient clinics (CBOCs).

At baseline, none of the study clinics had a PC-MHI program that met VA policy requirements to implement 1) a blend of co-located collaborative care and care management and 2) evidence-based care models (see Table 1). To prepare for program implementation, we required intervention clinics to identify existing staff or hire new staff who could serve as PC-MHI providers. Facilitators conducted the first site visit once clinics identified or hired appropriate staff. Four of eight IF clinics had co-located mental health providers in primary care prior to the intervention, but those providers were delivering specialty mental health services rather than the brief models of care associated with PC-MHI. One IF clinic had identified PC-MHI staff members who were co-located but providing only mental health assessments. Two other IF clinics had identified or hired providers for PC-MHI but none were providing PC-MHI services and one IF clinic had no PC-MHI staff. In the comparison networks, only one clinic had identified and hired PC-MHI staff but those staff members were not yet providing PC-MHI services. None of the other comparison clinics had designated PC-MHI staff.

**Table 1:** Status of study clinics’ PC-MHI program implementation at baseline (August 2009)

Site	Clinic Size (# of unique patients)	Staff Identified for PC-MHI	Policy-compliant PC-MHI Program?
Implementation Facilitation (Intervention) Sites			
Network A			
Site A1—VAMC	5632	MSW,^a^ psychiatrist^a^	No
Site A2—CBOC	9224	MSW^b^	No
Site A3—CBOC	4025	MSW^b^	No
Site A4—CBOC	5654	MSW^b^	No
Network C			
Site C1—VAMC	34,805	MSW^b^	No
Site C2—CBOC	14,763	MSW,^c^ RN^c^	No
Site C3—CBOC	8125	newly hired MSW^a^	No
Site C4—CBOC	4715	No^d^	No
Comparison Sites			
Network B			
Site B1—VAMC	7454	No^d^	No
Site B2—CBOC	11,308	No^d^	No
Site B3—CBOC	5944	No^d^	No
Site B4—CBOC	7527	No^d^	No
Network D			
Site D1—VAMC	35,000	RN,^e^ 2 newly hired psychiatrists^f^	No
Site D2—CBOC	13,600	No^d^	No
Site D3—CBOC	8463	No^d^	No
Site D4—CBOC	4527	No^d^	No

*VAMC* VA Medical Center, *CBOC* community-based outpatient clinic

^a^Personnel identified to fill PC-MHI position but not yet serving in that capacity

^b^Identified PC-MHI personnel co-located in primary care, providing specialty mental health care services

^c^Identified PC-MHI personnel co-located in primary care, conducting mental health assessments

^d^Not applicable, personnel have not yet been identified or hired for PC-MHI

^e^Identified PC-MHI personnel co-located in primary care, conducting triage and referral services

^f^Newly hired PC-MHI personnel co-located in primary care; not yet providing services

### The implementation facilitation strategy

The multifaceted IF strategy that we evaluated incorporated a blend of external and internal facilitation [40]. The national expert facilitator (JEK) worked with and mentored two internal regional facilitators who were clinical personnel experienced in PC-MHI practices. Each of the regional facilitators worked on the project for 50% of their time. Below we describe the facilitation activities they performed.

In preparation for helping sites implement PC-MHI, the facilitators began engaging clinic leaders and provided information about PC-MHI programs as well as what to expect during the facilitation process. Facilitators used formative evaluation techniques [47] to assess clinics’ current practice patterns and anticipate barriers and facilitators to PC-MHI implementation. They also identified key stakeholders who should participate in designing an implementation plan (see below) to adapt the PC-MHI program to meet site needs, preferences and priorities for local implementation. Facilitators obtained additional site level information from administrative data (e.g., number of patients seen in primary care) and medical center webpages (e.g., organizational charts).

The facilitators conducted site visits in August and September 2009 to begin guiding local teams in designing their implementation plans. Prior research [40, 48–51] informed site visit agendas and activities designed to engage key stakeholders in implementation planning processes. During site visits, the expert facilitator conducted academic detailing [52] for leaders and other stakeholders and assessed clinics’ readiness to plan the implementation process. She then held a final leadership briefing during which she provided her assessment of the clinic’s ability to implement a PC-MHI program and discussed barriers and facilitators to implementation identified to date. This was an important activity in the implementation planning process because it provided facilitators an early opportunity to identify potential problems and work directly with leaders to develop solutions to address implementation barriers. In addition to the academic detailing provided during the initial site visit, the facilitators conducted education and marketing activities throughout the intervention, providing ongoing training, engaging stakeholders and helping sites identify and resolve problems.

**Fig. 1:**
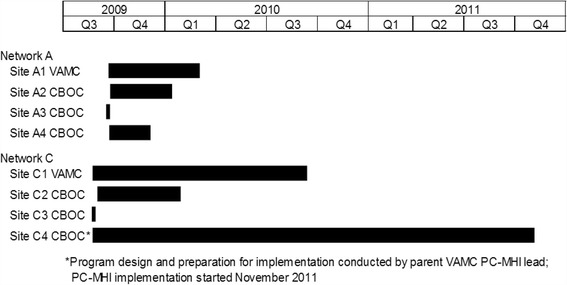
Length of time before sites were able to create implementation plans. *Bars* indicate the period of time during which facilitators helped intervention sites prepare to develop a PC-MHI program implementation plan. The beginning of the *bar* indicates facilitators’ first site visit. The end of the *bar* signals the completion of this plan. Two sites, A3 and C3, completed the plan during the first site visit

Once facilitators determined that a site was ready to begin in-depth planning, they used a checklist [53] to guide the decision-making process (e.g., identifying PC-MHI target population and exclusion criteria, PC-MHI staff members’ roles and responsibilities, and how to monitor the implementation process). Facilitators documented specific action items and, when appropriate, identified parties responsible for each action item. Although the facilitators initially intended to conduct this planning process during the first site visit, only two sites were ready at that time. At the other six sites, facilitators continued working with teams until they were ready to develop an implementation plan (see Fig. 1). It took five of the remaining six sites from a month to approximately a year to become ready to complete the implementation planning checklist. Site C4, a CBOC whose parent VAMC did not have a PC-MHI program, proved particularly challenging. After 6 months of working with this site, facilitators determined that without resources and support from the VAMC, this site would be unable to implement PC-MHI. Facilitators then provided support and consultation to the parent VAMC, which successfully implemented its own program and in November 2011, started implementing a PC-MHI program at site C4.

Once sites created their program plans, they could begin implementation of the plan. Facilitators monitored the implementation process, utilizing administrative data and assessing site progress in completing action items and achieving performance targets specified in the local implementation plan. The internal regional facilitator worked individually with each site to address site level needs such as additional provider education on the PC-MHI model and how to implement it, refinement of the site’s implementation plan, and marketing the potential outcomes and value of the PC-MHI program to primary care providers, Veterans and leadership. The external expert/internal regional facilitator pair in each network also established a learning collaborative [54] of IF site PC-MHI providers and champions to review implementation progress, share lessons learned, identify barriers and facilitators and create potential solutions to implementation problems.

Facilitators helped sites integrate the PC-MHI program into existing clinical programs and services, as well as continued problem identification and resolution and audit and feedback of the implementation process. Further, the internal regional facilitator continued to engage local leadership through regular briefings and email communication. Over the course of the implementation process, the expert facilitator mentored the internal regional facilitator in implementation strategies, transferring the knowledge and skills necessary to support ongoing program sustainment. Thus, these skills were retained within the IF networks once project funding ended. It is important to note that though we have described facilitation activities in a linear phasic framework, the IF process was actually dynamic and iterative, with activities overlapping and repeating to continually monitor and adjust local implementation processes to maximize potential for success [41, 42].

### Measure development

Assessing program implementation typically involves assessing fidelity, or the degree to which a program is implemented as its developers intended [55–58]. Scholars suggest that fidelity should be assessed using measures of adherence to a program, dose or amount of the program that is delivered, quality of program delivery, participant responsiveness and program component differentiation [59]. A description of a program’s components (strategies, activities, behaviors, media products and technologies, intended recipients and delivery settings) is the ideal basis for measuring the extent of implementation [60]. However, we faced a number of challenges to conducting a traditional fidelity assessment of the extent of PC-MHI implementation in this study. VA policy mandated implementation of a “blend” of two different types of PC-MHI care, co-located collaborative care and care management, both of which required significant structural and procedural changes in the delivery of mental health care. Further, although facilities were expected to implement evidence-based care models, there are many models that fit this criterion [61, 62]. Specific model selection was left to the discretion of the VA facilities. Additionally, given the variability in resources, structures and processes across VA, facilities also needed to make adaptations to their local contexts. Because there was not a clearly defined list of required program components and VA administrative data did not include quantitative measures of program components, we developed a holistic qualitative assessment instrument to document the PC-MHI program components each site implemented.

We developed the PC-MHI Program Component Assessment instrument using several inter-related sources. The first source was a set of quality improvement (QI) tools that LEP created for facilities to self-assess their fidelity with three different PC-MHI models of care, Translating Initiatives in Depression into Effective Solutions (TIDES) [37], the Behavioral Health Laboratory (BHL) [63], and Co-located Collaborative Care (CCC) [32]. To develop these tools, she collaborated with VA’s PC-MHI model developers, conducting in-depth interviews with them and reviewing program materials (e.g., training manuals and assessment tools), journal articles and conference presentations describing the models’ goals and outcomes. She then iteratively revised these tools with the model developers. The second source was VA’s first national “VAMC Primary Care – Mental Health Integration Survey.” The PC-MHI National Evaluation Team, charged with assessing implementation progress across VA, needed an instrument to assess PC-MHI program implementation. Although they were able to capture some measures of implementation outcomes through VA administrative data, there was no data available that could measure if PC-MHI was being fully implemented. As described above, because of the complex multifaceted nature of PC-MHI programs and the large number of variations that would meet policy requirements for implementing evidence-based programs, it was not possible to develop quantitative implementation measures. Thus the VAMC survey was a holistic qualitative measure. To develop that instrument, LEP and MJR collaborated with VA’s PC-MHI National Evaluation Team to combine the QI fidelity tools into a single self-administered survey. To create the PC-MHI Program Component Assessment instrument for the current study, we modified the national PC-MHI survey for telephone administration, retaining the closed response set but including opportunities for informants to clarify their answers. Our resulting PC-MHI Program Component Assessment instrument (see Additional file 1) addresses the following domains:

- Staffing issues including: PC-MHI occupations and availability of care managers, co-located prescribers and therapists.
- Mental health conditions addressed.
- Mental health services provided.
- Program policies and procedures including those for referral and length and number of sessions available, suicide prevention, program monitoring, evaluation and quality improvement.
- Types of assessment and communication tools utilized.
- Extent of communication between primary care and mental health services providers and staff members.
- Types of PC-MHI training available to providers and staff members.

### Data collection: site programs

Internal regional facilitators at IF sites and VAMC and network mental health leaders for comparison sites identified study sites that had PC-MHI programs and the VA clinicians providing services or the leaders for those programs, i.e., those responsible for implementing or overseeing implementation of PC-MHI program components. Only sites with PC-MHI programs had PC-MHI service providers or leaders. To recruit participants, facility mental health leaders sent letters to site providers and/or leaders briefly describing the study and introducing them to the study team. We followed up with an email asking if they would be interested in learning more about the study and possibly participating in it. We sent a letter containing the required elements of informed consent, including the study’s purpose and procedures, privacy protections and the risks, benefits and voluntary nature of participation to those who were interested. We recruited a total of twenty-six unique individuals; two of those declined participation for a total study *N* = 24 (see Table 2).

**Table 2:** Program component assessment (PCA) study sites and informants

Site	Initial PCA Informant (s) (*n* = 12) ^c^	Final PCA Informant (s) (*n* = 17) ^c^
Implementation Facilitation (Intervention) Sites		
Network A		
Site A1—VAMC	Internal Regional Facilitator	1 PC-MHI provider^a^
Site A2—CBOC	1 PC-MHI provider	2 PC-MHI providers^a^
Site A3—CBOC	1 PC-MHI provider	1 PC-MHI provider
Site A4—CBOC	1 PC-MHI provider	1 PC-MHI provider
Network C		1 centralized PC-MHI provider
Site C1—VAMC	1 PC-MHI provider	1 PC-MHI provider^a^
Site C2—CBOC	2 PC-MHI providers	1 PC-MHI provider^a^
Site C3—CBOC	1 PC-MHI provider	1 PC-MHI provider^a^ and 1 Internal Regional Facilitator^a^
Site C4—CBOC	No PC-MHI program	1 VAMC PC-MHI Leader
Comparison Sites		
Network B		
Site B1—VAMC	No PC-MHI program	1 PC-MHI provider
Site B2—CBOC	2 PC-MHI providers	1 PC-MHI provider
Site B3—CBOC	1 PC-MHI provider	1 PC-MHI provider
Site B4—CBOC	1 PC-MHI provider	2 PC-MHI providers (1 was different^a^)
Network D		
Site D1—VAMC	^b^	1 PC-MHI provider/leader
Site D2—CBOC	No PC-MHI program	No PC-MHI program
Site D3—CBOC	No PC-MHI program	No PC-MHI program
Site D4—CBOC	No PC-MHI program	No PC-MHI program

*VAMC* VA Medical Center, *CBOC* community-based outpatient clinic

^a^Different informant (s) than initial PCA informants

^b^No Program Component Assessment was conducted

^c^Total study *N* = 24 *unique* participants

One year after IF clinics with PC-MHI programs completed the initial program design and implementation plan and approximately 1 year after that, we administered the Program Component Assessment (PCA) instrument to IF site participants (*N* = 17) during one-hour telephone interviews. At comparison sites with PC-MHI programs, we conducted the interviews with study participants (*N* = 7) at approximately the same time as their matched IF site. Two VA health services researchers with expertise in qualitative data collection and analysis conducted all interviews. Interviewers had no relationship with the participants before the study began. At the beginning of the interviews, the interviewers introduced themselves as VA researchers testing an innovative network-level facilitation method within the context of the VA’s requirements for integrating mental health services into primary care settings, reviewed the elements of consent in the letter participants had received, and obtained verbal informed consent. The primary interviewer (MJR), a female licensed clinical social worker and PhD candidate in Public Policy, administered the PCA, clarifying questions and responses. The second interviewer, a male who earned his DHSc in Health Sciences Leadership and Organizational Behavior during the project and who was also Project Coordinator, requested additional clarification as needed. Both interviewers documented informant responses, took notes on information that did not fit into the existing response set, and documented questions for which informants provided information that might need to be reviewed later. We audio-recorded all interviews. Interviewers met immediately after the interview to confirm responses and compare notes.

Because the PCA interviews created a large amount of raw data, we developed a structured summary template to facilitate comparison and rating of sites’ programs. The template used bulleted lists, tables and checkboxes to display program information so that it could be more easily reviewed (see Additional file 2). Utilizing this template, we then created structured summaries describing the components of the PC-MHI program for each site and time period. In addition to information that we obtained from the PCA interviews, the summaries also included information about clinic size and the availability of mental health specialty care services at the start of the study. We prepared a total of twenty-three program summaries for the sites that had PC-MHI programs (10 sites at the initial assessment and 13 at the final assessment). In preparation for the expert rating process described below, we removed study site identifiers and the time period of the PCA from the summaries.

### Data collection and analysis: expert ratings of site programs

To assess how well sites implemented PC-MHI programs, VA PC-MHI experts rated the PCA summaries. Experts have an extensive body of domain knowledge organized abstractly in “mental models” of how things work [64, 65]. They also have refined reasoning processes that allow them to perceive and interpret complex patterns in a set of information, to detect problems or anomalies and to run mental simulations to predict how things will work in the future [65, 66]. Thus, we expected that VA PC-MHI experts would be able to review and assess each unique PC-MHI program based on the Program Component Summary we provided them. We recruited six national experts in PC-MHI care models and evidence-based interventions, as well as VA policy, organizational structures and processes, to rate the programs. Experts reviewed the program summaries and rated them on four dimensions: the extent to which the program made use of evidence-based strategies for improving mental health care within primary care, the overall program quality, the potential for long-term sustainability, and the likely level of improvement in quality of care the intervention would produce. Experts reviewed a mix of initial and final PCA summaries across IF and comparison sites and did not review the same site more than once. Following Parker and Rubenstein [48, 67], experts were blind to informant, site and time period and made their ratings independently using 7-point Likert scales. For each program summary, we collected ratings from a total of 3 reviewers. Because each expert had to review eleven or twelve program summaries and rate each one on four dimensions, a substantial time burden, we did not ask experts to explain their ratings. We calculated average ratings for each program summary on rated dimensions. Because of our small sample size, we did not have sufficient power to conduct statistical analyses. We therefore conduct a qualitative assessment.

Our intervention sites began receiving IF before we could finalize selection of comparison sites and Institutional Review Board approval in one of the networks. We were therefore unable to collect initial program component data at one comparison network (D). We were, however, able to determine whether sites in this network did or did not have programs. As only one out of four did during the initial period, we were in fact, missing initial expert ratings at one site. We were able to obtain data to rate for all networks for the second rating period.

## Results

We found evidence supporting our hypothesis that facilitation could foster implementation of PC-MHI in VA primary care clinics. First, all sites receiving IF implemented PC-MHI programs but not all comparison sites were able to implement programs. At the time of the initial program assessment for each site, seven of eight IF sites and four of eight comparison sites had implemented a PC-MHI program (see Table 3). At the time of the final program assessment, 1 year later, all eight IF but only five comparison sites had implemented a PC-MHI program (see Table 4).

**Table 3:** Mean initial program ratings for intervention and comparison sites

Site	Overall Quality	Evidence	Sustainability	Level of Improvement
Intervention Sites – Network A				
Site A1 – VAMC	3.33	3.33	3.33	3.33
Site A2 – CBOC	4.33	4.67	4.00	4.33
Site A3 – CBOC	4.67	5.00	5.00	4.33
Site A4 – CBOC	4.00	4.00	3.67	4.00
Intervention Sites – Network C				
Site C1 – VAMC	4.00	3.33	3.67	4.33
Site C2 – CBOC	1.33	1.33	2.00	2.00
Site C3 – CBOC	6.33	6.33	6.00	6.33
Site C4 – CBOC	No PC-MHI program	No PC-MHI program	No PC-MHI program	No PC-MHI program
Comparison Sites – Network B				
Site B1 – VAMC	No PC-MHI program	No PC-MHI program	No PC-MHI program	No PC-MHI program
Site B2 – CBOC	2.67	2.67	5.33	3.67
Site B3 – CBOC	2.33	2.33	2.00	2.33
Site B4 – CBOC	1.33	1.33	2.00	2.00
Comparison Sites – Network D				
Site D1 – VAMC	^a^	^a^	^a^	^a^
Site D2 – CBOC	No PC-MHI program	No PC-MHI program	No PC-MHI program	No PC-MHI program
Site D3 – CBOC	No PC-MHI program	No PC-MHI program	No PC-MHI program	No PC-MHI program
Site D4 – CBOC	No PC-MHI program	No PC-MHI program	No PC-MHI program	No PC-MHI program

*VAMC* VA Medical Center, *CBOC* community-based outpatient clinic

Programs were rated on a scale from 1 to 7 with 1 = Low and 7 = High

^a^Missing data – unable to conduct program component assessment data

**Table 4:** Mean final program ratings for intervention and comparison sites

Site	Overall Quality	Evidence	Sustainability	Level of Improvement
Intervention Sites – Network A				
Site A1 – VAMC	5.00	4.33	5.33	5.00
Site A2 – CBOC	5.33	5.33	5.00	5.33
Site A3 – CBOC	5.33	5.67	5.33	5.67
Site A4 – CBOC	5.00	5.00	5.00	6.00
Intervention Sites – Network C				
Site C1 – VAMC	1.67	1.67	2.00	2.00
Site C2 – CBOC	3.00	3.00	3.00	2.67
Site C3 – CBOC	6.00	5.67	4.67	6.33
Site C4 – CBOC	3.67	3.33	4.67	3.67
Comparison Sites – Network B				
Site B1 – VAMC	2.00	2.33	3.33	3.33
Site B2 – CBOC	3.33	3.33	3.33	4.00
Site B3 – CBOC	3.67	3.33	5.00	3.67
Site B4 – CBOC	4.33	4.00	4.33	4.33
Comparison Sites – Network D				
Site D1 – VAMC	4.33	4.33	4.33	4.00
Site D2 – CBOC	No PC-MHI program	No PC-MHI program	No PC-MHI program	No PC-MHI program
Site D3 – CBOC	No PC-MHI program	No PC-MHI program	No PC-MHI program	No PC-MHI program
Site D4 – CBOC	No PC-MHI program	No PC-MHI program	No PC-MHI program	No PC-MHI program

Programs were rated on a scale from 1 to 7 with 1 = Low and 7 = High

*VAMC* VA Medical Center, *CBOC* community-based outpatient clinic

We were also interested in exploring whether IF could foster the development of high quality PC-MHI programs that adhere to evidence, are sustainable and are likely to lead to improvement of clinical practices and outcomes. At the time of the initial program assessment, experts rated IF programs higher than those at comparison sites with one exception. Program ratings for IF site C2 were very low. At the time of the final assessment, experts again rated IF site programs higher than their matched comparison sites with one exception. Site C1 was rated lower on all four dimensions than all other IF and comparison sites. At both time points, ratings for each site were generally similar across all four dimensions. In comparing the initial and final ratings, all ratings improved over the course of the study in five of the seven IF sites and two of the three comparison sites.

## Discussion

Our qualitative analysis suggests that sites with challenging clinical contexts receiving the IF strategy were more likely to implement a PC-MHI program than were comparison sites. Indeed, whereas all the intervention sites implemented programs, some of comparison sites never did during the study period. Our analysis also suggests that IF is effective in fostering clinical practice quality as well as adherence to the evidence for PC-MHI at sites that lack knowledge, skills and resources for implementation. Further, during the second year of implementation, most sites receiving IF were not only able to sustain but actually able to continue improving program quality and adherence to evidence. These findings complement our findings from another project study that this strategy increases the likelihood that primary care providers will adopt the program, collaborating with and referring to PC-MHI providers [43]. Interestingly, in that other study, we did not find a difference in our quantitative fidelity measure (same day access). That measure only assessed one aspect of PC-MHI. PC-MHI, however, is extremely complex, with many interacting components and multiple evidenced-based methods with multiple acceptable implementation methods. As such, assessing its fidelity requires an in-depth program assessment beyond what one single quantitative measure can provide. Our study provides such an in-depth assessment. Additionally, our findings here are consistent with the growing body of evidence showing that facilitation improves the uptake of evidence-based practices and programs [25, 68, 69].

Our IF strategy appears to be effective in part because facilitators applied evidence-based implementation science strategies in their efforts to help sites implement PC-MHI. For example, at intervention sites, facilitators persistently sought to involve stakeholders across all organizational levels [49] and engage them in a QI dialogue [50]. It is likely that there was no one to do this at the three comparison sites that still had not implemented PC-MHI by the end of the study. Facilitators also assessed the organizational context and current practices and tailored implementation efforts to site context and needs, activities that evidence suggests will improve implementation [25, 70, 71]. We attribute the relatively high IF site program quality and adherence to evidence to facilitators’ efforts to 1) help site staff acquire the requisite knowledge and skills for delivering high quality PC-MHI care using evidence-based strategies, and 2) monitor programs and intervene to prevent program “drift,” e.g., by providing feedback to site stakeholders [58].

Despite the application of evidence-based implementation strategies, some of our study sites in IF Network C still had difficulty implementing PC-MHI. Site C1 was a VAMC primary care clinic and site C2 was the CBOC associated with that VAMC. Facilitators were unable to engage high-level leaders’ support at the parent VAMC, limiting resources available for needed structural change at both sites. However, with the facilitators’ assistance, site C1 was able to implement a program with quality and adherence to evidence ratings similar to other intervention sites by the end of the first year. Nevertheless, there was significant PC-MHI staff instability in the 6 months prior to our final assessment. At the time of our assessment, the new PC-MHI provider had just started; low ratings likely reflected this. Site C2 had low ratings at the time of the initial assessment. In addition to lack of high-level leadership support at the VAMC level, neither of the clinic leaders was engaged at the time of the first assessment and one of them was very resistant to change. PC-MHI provider time was diverted to conducting lengthy mental health assessments and referrals. The first set of ratings for this site reflects these circumstances. With intensive assistance, facilitators were able to help site C2 improve program quality and adherence to the evidence but only on a limited basis. Lack of VAMC support was also a challenge for helping implement PC-MHI at site C4. However, facilitators’ efforts were successful in engaging the site’s parent VAMC leadership to support implementation by the end of the study. By the time of our second assessment, this site was able to implement a program of moderate quality. Thus although IF clearly fosters implementation, facilitators will have difficulty being successful unless they obtain at least a moderate level of leaders’ support, including provisions of basic resources.

**Fig. 2:**
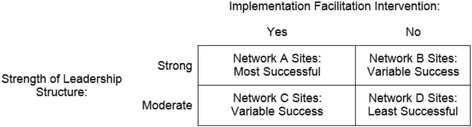
Combined effects of implementation facilitation and strength of leadership structure on implementation. Implementation facilitation and strength of leadership structure interact and affect how successful sites are in implementing evidence-based programs. Programs are most successful when there is a strong leadership structure and sites receive implementation facilitation

Implementation science scholars generally agree that the engagement and support of leadership at all levels is an important determinant of implementation success [8, 72–74]. In comparing all eight sites, it appears that the strength of network mental health service line structure influenced PC-MHI program implementation regardless of the initial engagement and support of leadership at lower levels. All sites in IF network A and comparison network B, which had strong network leadership structures, implemented programs and program quality and adherence to evidence improved over time. In IF clinics, facilitators engaged local leadership in network A and helped sites implement higher quality programs than comparison network B. In the two networks where the strength of mental health service line structures was relatively moderate, the results were variable. Only one site in IF network C implemented a high quality program and only one site in comparison network D implemented a PC-MHI program at all. Thus it appears that facilitation and strength of leadership structure may have a synergistic effect on ability to implement higher quality and evidence-based programs. Without either success is unlikely. With one or the other, success is possible but difficult and with both, success is most likely. See Fig. 2.

Although we cannot be sure why some comparison sites did better than others, one possible explanation concerns the level of commitment from the network mental health lead. As we described, our IF intervention required that clinics identify or hire PC-MHI staff. The mental health lead in the comparison network with a strong network structure had prior positive experience with PC-MHI. Other researchers have found that preexisting familiarity with the clinical intervention can foster adoption [14]. In our study, the network mental health lead in the comparison network with a strong network structure required clinics to assign staff for PC-MHI and clinics in this network were able to implement programs without IF assistance. Thus, IF might be most important for networks with weak leadership structures and support for programs. Future research should address this issue.

Future research should also explore whether there are other barriers to successful application of the IF strategy. Early identification of such barriers would allow researchers and/or clinical managers to delay facilitation efforts until barriers can be addressed, or to select sites that are more likely to benefit from IF. Finally, although the IF strategy we used was effective and built organizational capacity, it was also resource intensive. Given IF’s costs we recommend reserving it for only those sites most needing this assistance. Future research should develop tools for identifying those sites that are both able to avail themselves of IF assistance and that find implementation sufficiently difficult that it is worth the cost of intervening. Finding the balance will depend in part upon organizational priorities and resources.

In addition to improving PC-MHI program quality and adherence to evidence, the IF strategy we applied built capacity at the regional level for facilitating implementation of PC-MHI. At the beginning of the study, internal regional facilitators were IF novices. Over time, the expert facilitator mentored internal facilitators, helping them develop IF skills and handing off responsibility to them when they were ready. By the end of the study, internal facilitators were IF experts, applying evidence-based strategies and working to maintain and improve program quality and adherence to evidence. The IF skills they developed are readily transferrable to implementation of other evidence-based innovations. Implementation scientists generally seek to integrate evidence-based practices and programs into routine care by the end of a study. We recommend incorporating a process for handing off or transferring IF knowledge and skills to internal change agents. This could, as it did in this study, maximize potential for sustainment, continued improvement in quality and adherence to evidence, as well as build capacity for implementing other innovations. Although there is currently limited information regarding how to transfer IF knowledge and skills [29], another part of this project has begun to document this process [42].

### Limitations

Although we believe that our study demonstrates that IF has great promise for helping challenged clinics to implement evidence based clinical programs, there were limitations. First, as we described earlier, we were unable to randomize to condition. Second, resources available for the study limited its scope. For example, we have less data on the comparison than on the intervention clinics. There may have been factors that we were unable to assess that could have contributed to or hindered adoption of PC-MHI at comparison clinics. Also, due to resource limitations, we were unable to involve more than eight intervention and eight comparison sites, thus limiting our ability to conduct statistical analyses on rating data. Additionally, we were only able to interview the people who knew the most about the PC-MHI program and services at each site, generally PC-MHI staff. It may have been beneficial to seek the perspectives of other stakeholders, including other clinicians and consumers. However, given the already broad scope of this study, we lacked resources to query additional stakeholders. A final study limitation is that we did not ask experts to give reasons for their ratings. We felt that the time burden for experts outweighed any potential benefits of additional explanation. Despite these limitations, we are confident that our evaluation and the tools we developed to assess implementation of these very complex programs should prove useful to others, both within and outside of VA, who are implementing and evaluating similarly complex, real-world quality improvement efforts.

## Conclusions

Implementing complex evidence-based programs, particularly in settings that lack infrastructure, resources and support for such efforts, is challenging. We found that a blend of external expert and internal regional facilitation incorporating implementation science improved PC-MHI program uptake, quality and adherence to evidence in primary care clinics with these challenges. This study was conducted within the context of a VA policy initiative that required sites to implement a blend of two different, complex PC-MHI care models. The methods we developed for measuring program quality and adherence to evidence addressed this complexity.

Our findings have implications for research using implementation facilitation strategies, as well as for clinical initiatives implementing evidence-based interventions. In fact, VA leadership has already adopted this facilitation strategy to support implementation of PC-MHI, as well as other policy-mandated evidence-based innovations, at facilities across VA that struggle to implement new programs [9, 10, 15, 75]. Based on experiences of facilitators in this study, we developed IF training materials and the external expert facilitator (JEK) and one of the internal regional facilitators have been training clinical operations personnel in these methods [76, 77]. They have also trained other researchers in the application of implementation facilitation. Scholars have long advocated that implementing evidence-based practices and programs will improve healthcare quality and outcomes but clinical settings continue to struggle with implementation challenges. The IF strategy we describe enhances sites’ ability to successfully implement such innovations.

## Additional files

Additional file 1: PC-MHI Program Component Assessment interview guide. Assessment instrument/interview guide for obtaining and documenting information about the components of each facility’s program for integrating mental health services into primary care. (DOCX 102 kb)

Additional file 2: PC-MHI program components summary template. Template for creating a summary of the components of each facility’s program for integrating mental health services into primary care. Designed for use with the “PC-MHI Program Component Assessment Interview Guide.” (DOCX 52 kb)

## Abbreviations

BHL: Behavioral Health Laboratory
CBOC: Community based outpatient clinic
CNE: Carrie N Edlund
IF: Implementation facilitation
i-PARIHS: Integrated-Promoting Action on Research Implementation in Health Services
JEK: JoAnn E Kirchner
LEP: Louise E Parker
MJR: Mona J Ritchie
MSW: Master of social work
PARIHS: Promoting action on research implementation in health services
PCA: Program Component Assessment
PC-MHI: Primary Care-Mental Health Integration
QI: Quality improvement
RN: Registered nurse
TIDES: Translating initiatives for depression into effective solutions
VA: Department of Veterans Affairs
VHA: VA Medical Center
VAMC: Veterans Health Administration
