# Supplementary material for: Using implementation facilitation to foster clinical practice quality and adherence to evidence in challenged settings: a qualitative study
Source: BMC Health Serv Res. 2017 Apr 20;17:294. doi: 10.1186/s12913-017-2217-0 (PMC5397744; doi:10.1186/s12913-017-2217-0)
Supplement: Supplementary file 1 — PC-MHI Program Component Assessment interview guide. Assessment instrument/interview guide for obtaining and documenting information about the components of each facility’s program for integrating mental health services into primary care. (DOCX 102 kb) [file 12913_2017_2217_MOESM1_ESM.docx]

**Primary Care-Mental Health Integration (PC-MHI)**

**Program Component Assessment Interview Guide**

Purpose

The purpose of the assessment instrument is to gain a comprehensive understanding of each facility’s program for integrating mental health into primary care.

Informant Selection

Select and interview the individuals(s) most likely to be able to describe the program (e.g., a PC-MHI provider, PC-MHI champion, clinical leader). If that person is unable to respond to all the component assessment items, use snowball sampling to select other participants who can complete the assessment. Numbers and types of informants will vary by site.

Specific Instructions

Use one assessment form to document program components for each facility, regardless of the number of individuals you interview. Note: The *PC-MHI Program Components Summary Template* can be used in conjunction with this Interview Guide to summarize program data for each facility.

Many items have multiple formats to take into the account the fact that 1) informants may be in different positions and 2) you may be asking about a single person or multiple persons depending on the answers to previous questions. Select the text that is most appropriate for the person you are interviewing and about whom you are asking questions.

If participants do not know the answer or refuse to answer, write DNK by the question on the survey form. If you do not ask that question, write DNA by the question on the survey form.

Instrument Authors:

Louise E. Parker, Ph.D. and Mona J. Ritchie, PhD, MSW

The authors are grateful to the following (listed alphabetically) for their input and source materials concerning the content and fidelity assessment of PC-MHI programs:

Edmund Chaney, PhD, Brady H. Cole, MA, MPH, Johanna Klaus, PhD, John F. McCarthy, PhD, MPH,  David Oslin, MD, Andrew Pomerantz, MD, and Lisa V. Rubenstein, MD, MSPH.

For information about this instrument and how to utilize it, please contact:

Mona J. Ritchie, PhD, MSW

Implementation Coordinator

VA QUERI Program for Team-Based Behavioral Health

Phone: (501) 257-1735

Email: [Mona.Ritchie@va.gov](mailto:Mona.Ritchie@va.gov)

Today we will be focusing on what your clinic is doing to meet the VA’s requirements for integrating mental health care. By integrated care, we mean utilizing care managers and/or co-located mental health staff members who help clinics to manage mental health needs within primary care rather than through a traditional specialty mental health service. (*This introduction can be adapted for use outside of VA.)*

**Section 1: Staff and Conditions Addressed Overview**

1. **Staff Overview**

**Introduction for Programs with Staff (Version 1):**

**For integrated care provider informants**: Including yourself, do any of the following types of staff members work on integrated care at your clinic?

**For non-integrated care provider informants:** Do any of the following types of staff members work on integrated care at your clinic?

**Introduction for Planned Programs with Planned Staff (Version 2):**

**For integrated care provider informants**: Including yourself, will any of the following types of staff members work on integrated care at your clinic?

**For Non-integrated care provider informants:** Will any of the following types of staff members work on integrated care for your clinic? Some integrated care staff may be located physically at your clinic and others may be at another location, for example, some clinics share a care manager.

| **Check all that apply:** | **Existing Staff** | | **Planned Hires** |
| --- | --- | --- | --- |
|  | Number ICPs  (e.g., 2 ½ time = 2) | Total ICP FTE  (e.g., 2 ½ time = 1 FTE) | Check if Planned only |
| 1. Psychiatrists |  |  | 1.  |
| 1. Prescribing Advance Practice Nurses such as a Nurse Practitioner with expertise in mental health issues |  |  | 1.  |
| 1. MH Masters Level Social Workers or MSWs with expertise in mental health issues |  |  | 1.  |
| 1. PhD Level Psychologists |  |  | 1.  |
| 1. Masters Level Psychologists or other type of master’s level counselor |  |  | 1.  |
| 1. Physician Assistants or PAs with expertise in mental health |  |  | 1.  |
| 1. Registered Nurses or RNs without advanced training |  |  | 1.  |
| 1. Non-RN Bachelors Level personnel such as technicians |  |  | 1.  |
| 1. Clerks |  |  | 1.  |
| 1. Other1; 2. Describe________________ |  |  | 1.  |
| 1. Other2; 2. Describe_______________ |  |  | 1. ­­­ |
| 1. Other3; 2. Describe_______________ |  |  | 1.  |
| 1. Other4; 2. Describe______________ |  |  | 1.  |
| 1. Other5; 2. Describe_______________ |  |  | 1.  |
| 1. Other6; 2. Describe_______________ |  |  | 1.  |

1. I am going to read you a list of conditions. Please tell me whether you address these (**OR** your clinic’s integrated care staff members address these **OR** your integrated care staff member addresses these):

| 1. **** Major Depression | | |
| --- | --- | --- |
| 1. **** Mild to moderate depression | | |
| 1. **** Anxiety ***OR*** **S1_2d. ** Anxiety only when co-morbid with depression | | |
| 1. ****PTSD ***OR* S1_2f. **  PTSD only when co-morbid with   depression | | |
| 1. **** Alcohol misuse/abuse/heavy   drinking/problem drinking | ***OR*** | **S1_2h. ** Alcohol misuse/abuse/heavy  drinking/problem drinking only  when co-morbid with depression |
| 1. **** Alcohol dependence | ***OR*** | **S1_2j. ** Alcohol dependence only when co-morbid with depression |
| 1. **** Bipolar Disorder | | |
| 1. **** Schizophrenia | | |
| 1. **** Other      1. Describe:_______________________________________________ | | |

**Section 2: Co-Located Care Issues**

**Questions about prescribers:**

| 1. You mentioned that your clinic has (*list any of the following:* psychiatrists, APNs).   Is this psychiatrist/APN (or are any of these) co-located with your PC providers? | | **YES** | | **NO🡪skip to S 2_11** | |
| --- | --- | --- | --- | --- | --- |
| 1. *If have psychiatrist(s):* Is there a psychiatrist (or are there any psychiatrists) co-located in PC?    1. _____Number co-located    2. _____Total percentage/FTE co-located | | **YES** | | **NO🡪skip to S 2_3** | |
|  |  |  |  |  | |
| 1. *If have MH APNs:* Is there an APN (or are there any APNs) with prescriptive authority and mental health expertise co-located in PC?   If yes:   1. _____Number co-located 2. _____Total percentage/FTE co-located | | **YES** | | **NO🡪skip to S 2_4**  **OR**  **if NO to both S2_2 and S2_3, skip to S2_11** | |
| 1. Is/Are the co-located psychiatrist(s) (and/or) APN(s) office(s) interspersed with the PC provider offices (in other words, in same suite or hallway as those of PC providers)?    **YES, all are** (**skip to S2_6**)  **SOME are** (**skip to S_6**)  **NO, none are**   1. *If more than one prescriber*: Then which of the following would you say best describes where these offices are located? **[Interviewer asks all 3 together but only checks one]**  \|  **All** are located in the **same** building as the PC provider offices **OR**   **Some** are located in the same building as PC provider offices **OR**   **All** are located in a **different** building(s) than PC provider offices \| \| --- \| | | | | | |
| *If just one prescriber*: Then which of the following would you say best describes where this office is located? **[Interviewer asks both together but only checks one]** | | | | | |
| \|  **Office is** located in the **same** building as the PC provider offices **OR** \| \| --- \| \|  **Office is** located in a **different** building than PC provider offices \| | | | | | |
| 1. For most patients, is there immediate access to a/the co-located prescriber? In other words, patients typically do not have to wait at all. | | **YES** | | **NO** | |
| 1. If patients must wait to see a/the co-located prescriber, on average how long is their wait? |  20 minutes or less | |  21-30 minutes | |  > 30 minutes |
| 1. On average how long do patients spend with a/the co-located prescriber? |  20 minutes or less | |  21-30 minutes | |  > 30 minutes |
| 1. Is a/the co-located prescriber on duty whenever the primary care clinic is open? | | **YES** | | **NO** | |
| 1. **[Ask only if Yes to S2_3 (i.e., prescribing APN with MH expertise is co-located in PC)]**   Does (do) your clinic’s prescribing APN(s) have immediate access to an on-call psychiatrist? | | **YES** | | **NO** | |
| **Questions about therapists:** | | | | | |
| 1. You mentioned that your clinic has (*list any of the following: PhD psychologists, MSWs, MA level psychologists or counselors)*   Do any of these or any other type of provider serve as co-located therapists?  *Or if interviewing this person and is only one:*  Do you serve as a co-located therapist? | | **YES** | | **NO🡪skip to S2_19** | |
| 1. *If yes and there is more than one, determine type and number and FTEs co-located in primary care:* 2. **** Psychologists PhD level: 3. _____Number co-located 4. _____Total percentage/FTE co-located 5. **** MSWs: 6. _____Number co-located 7. _____ Total percentage/FTE co-located 8. **** Psychologists MA level or counselors: 9. _____Number co-located 10. _____ Total percentage/FTE co-located 11. **** Other: 12. Describe:_________________________________________ 13. _____Number co-located 14. _____ Total percentage/FTE co-located | | | | | |
| 1. Is the co-located therapist’s office (or are the co-located therapists’ offices) interspersed with PC providers (in other words, in same suite or hallway as PC providers)?    **YES, all are** (**skip to S2_15**)  **SOME are** (**skip to S2_15**)  **NO**   1. *If more than one prescriber:* Then which of the following would you say best describes where these are located? **[Interviewer asks all 3 together and checks one choice]**  \|  **All** are located in the **same** building as the PC provider offices **OR**   **Some** are located in the same building as PC provider offices **OR**   **All** are located in a **different** building(s) than PC provider offices \| \| --- \|   *If just one prescriber:* Then which of the following would you say best describes where this office is located? **[Interviewer asks both together but only checks one]**   \|  **Office is** located in the **same** building as the PC provider offices **OR**   **Office is** located in a **different** building than PC provider offices \| \| --- \| | | | | | |
| 1. For most patients, is there immediate access to a co-located therapist (in other words, patients do not have to wait at all)? | | **YES** | | **NO** | |
| 1. If a patient must wait to see a co-located therapist, on average how long is the wait? |  20 minutes or less | |  21-30 minutes | |  > 30 minutes |
| 1. What is the average amount of time patients spend with a co-located integrated care therapist? |  20 minutes or less | |  21-30 minutes | |  > 30 minutes |
| 1. Is a therapist on duty whenever the PC clinic is open and seeing patients? | | **YES** | | **NO** | |

**Questions about Communication**

*Ask the following two questions only if have co-located prescribers or therapists:*

1. Please rate your answer to following question on a scale of 1 to 7 where 1 is not at all, 4 is a moderate amount, and 7 is a great deal: To what extent do (the) co-located provider(s) and primary care providers communicate to incorporate patient preferences into treatment plans?

| **Not at all** |  | **A moderate amount** | | |  | **A great deal** |
| --- | --- | --- | --- | --- | --- | --- |
| 1 | 2 | 3 | 4 | 5 | 6 | 7 |
|  |  |  |  |  |  |  |

1. Using the same scale, to what extent do (the) co-located provider(s) and primary care providers communicate to develop and review treatment plans?

| **Not at all** |  | **A moderate amount** | | |  | **A great deal** |
| --- | --- | --- | --- | --- | --- | --- |
| 1 | 2 | 3 | 4 | 5 | 6 | 7 |
|  |  |  |  |  |  |  |

**Section 3: Care Management Issues**

| 1. Does the integrated care program include care management? By care management we mean, an integrated care provider who helps primary care providers manage mental health needs through such services as assessment and patient monitoring. | **YES** | **NO🡪skip to S4_1** |
| --- | --- | --- |
| 1. You mentioned that your clinic has (*list any of the follow*: Registered Nurse without advanced training, Advance Practice Nurse, Nurse Practitioner, MSW, Psychologist (MA or PhD), counselor, Pharmacist, Non-RN Bachelor’s level Personnel, Physician Assistant).   Do any of these (*or if only one integrated care staff member*: does the *fill in appropriate occupation*) or any other clinical staff members provide care management?  **YES🡪Complete S3_1b through S3_1f1**  **NO:** Then what type of professional or professionals provide care management for your clinic? **🡪 revise count of professions in section 1 overview and then🡪Complete S3_1b through S3_1f1**  *Determine the number and FTE care managers for each of the following:* | | |
| 1. ____Registered Nurse without advanced training 2. ____Total percentage/FTE care manager | | |
| 1. ____Advanced Practice Nurse (for example, NP) 2. ____Total percentage/FTE care manager | | |
| 1. _____Master of Social Work/MSW 2. ____Total percentage/FTE care manager | | |
| 1. _____Non- RN Bachelor’s-Level personnel 2. _____Total percentage time/FTE care manager | | |
| 1. _____Other; describe:______________________________________ 2. _____Total percentage time/FTE care manager | | |
| 1. Does your clinic’s care manager (Do your clinic’s care managers) typically contact patients by telephone rather than face-to-face? | **YES** | **NO** |
| *If there is only one integrated care provider and you have determined that person also serves as a co-located prescriber and/or therapist then***🡪Skip to S3_7** | | |
| 1. Is the care manager (are any care managers) located at this clinic? | **YES** | **NO🡪skip to S3_7** |
| 1. Is the care manager’s office (are the care managers’ offices) interspersed with PC providers (in other words, in same suite or hallway as PC providers)?    **YES, all are** (**skip to 6**)  **SOME are** (**skip to 6**)  **NO** | | |
| 1. *If more than one care manager*: Then which of the following would you say best describes where these are located? **[Interviewer asks all 3 together and checks one choice]**  \|  **All** are located in the **same** building as the PC provider offices **OR** \| \| --- \| \|  **Some** are located in the same building as PC provider offices **OR** \| \|  **All** are located in a **different** building(s) than PC provider offices \|   *If one integrated care provider*: Then which of the following would you say best describes where this office is located? **[Interviewer asks both together but only checks one]**   **Office is** located in the **same** building as the PC provider offices **OR**   **Office is** located in a **different** building than PC provider offices | | |
| 1. Does the care manager (Do any of the care managers) also serve as a co-located therapist? | **YES** | **NO** |
| 1. *Only ask if care manager is a prescriber (e.g.. is an APN, PA, MD)*   Does the care manager (Do any of the care managers) also serve as a co-located prescriber? | **YES** | **NO** |

1. I am going to read you a list of disorders. Please indicate if the care manager(s) handle(s) these within Primary Care and without referring to MH specialty care.

| 1. **** Major Depression | | |
| --- | --- | --- |
| 1. **** Mild to moderate depression | | |
| 1. **** Anxiety ***OR*** **S3_8d. ** Anxiety only when co-morbid with depression | | |
| 1. ****PTSD ***OR* S3_8f. **  PTSD only when co-morbid with depression | | |
| 1. **** Alcohol misuse/abuse/heavy drinking/problem drinking | ***OR*** | **S3_8h. ** Alcohol misuse/abuse/heavy drinking/problem drinking only when co-morbid with depression |
| 1. **** Alcohol dependence | ***OR*** | **S3_8j. ** Alcohol dependence only when co-morbid with depression |
| 1. **** Bipolar Disorder | | |
| 1. **** Schizophrenia | | |
| 1. **** Other: 2. describe:__________________________________________________ | | |

| 1. When care managers work (When the care manager works) with patients, do primary care providers always remain involved? | **YES** | **NO** |
| --- | --- | --- |
| 1. Approximately what percentage of your clinic’s primary care staff have met with the care manager (managers) either face-to-face or via video conferences? |  Less than 80% |  80% or greater |

1. How often does your clinic’s care manager (do your clinic’s care managers) receive supervision? **[can only pick one]:**

| Once a week for approximately an hour or more **OR** |
| --- |
| Less than once a week  **OR** |
| Once a week for less than an hour  **OR** |
| Never **[If never, skip to S3_13]** |

1. Who conducts this supervision? (**respondent can pick more than one):**

| 1. **** Psychiatrist |
| --- |
| 1. **** Advanced practice mental health nurse |
| 1. **** Psychologist |
| 1. **** Primary care physician |
| 1. **** Other: 2. If other, describe:____________________________________________ |

| 1. Does your clinic’s care manager (Do your clinic’s care managers) have access to CPRS Mental Health Assistant? | **YES** | **NO** |
| --- | --- | --- |
| 1. If your clinic’s care manager determines (If your clinic’s care managers determine) that a patient is at risk for suicide, does the care manager (do the care managers) have a way to contact appropriate personnel (for example, two phones lines; direct access to curbside consult)? | **YES** | **NO** |
| 1. If your clinic’s care manager determines (If your clinic’s care managers determine) that a patient is at risk for suicide is there always a back-up mental health provider trained to deal with suicide prevention on call? | **YES** | **NO** |

1. Please rate your answer to following question on a scale of 1 to 7 where 1 is not at all, 4 is a moderate amount, and 7 is a great deal: To what extent do care managers (does the care manager) and primary care providers communicate to incorporate patient preferences into treatment plans?

| **Not at all** |  | **A moderate amount** | | |  | **A great deal** |
| --- | --- | --- | --- | --- | --- | --- |
| 1 | 2 | 3 | 4 | 5 | 6 | 7 |
|  |  |  |  |  |  |  |

1. Using the same scale of 1 to 7: To what extent do care managers (does the care manager) and primary care providers communicate to develop and review treatment plans?

| **Not at all** |  | **A moderate amount** | | |  | **A great deal** |
| --- | --- | --- | --- | --- | --- | --- |
| 1 | 2 | 3 | 4 | 5 | 6 | 7 |
|  |  |  |  |  |  |  |

**Section 4: Program Activities**

**For integrated care provider informants**:

I am going to read a list of activities that you and/or other members of your clinic’s integrated care clinical staff might provide. Please tell me if your clinic’s integrated care staff members provide any of these services.

*If only one*: I am going to read a list of activities that you might provide. Please tell me if you provide any of these services.

**For non-integrated care provider informants:**

I am going to read a list of activities that your clinic’s integrated care clinical staff members might provide. Please tell me if your clinic’s integrated care staff members provide any of these services.

*If only one*: I am going to read a list of activities that your clinic’s [*list integrated care professions: e.g., your clinic’s integrated Social Worker]* provides. Please tell me if your clinic’s [*list integrated care professions: e.g., your clinic’s integrated Social Worker]* provides any of these services

| 1. **** Mental health assessment using formal tools such as the PHQ-9 | | |
| --- | --- | --- |
| 1. **** Ongoing monitoring of patient progress | | |
| 1. **** Clinical psychiatric evaluation without formal assessment tools | | |
| 1. **** Facilitating or providing advice about referrals to mental health specialty care | | |
| 1. **** Direct referral and/or transfer to mental health specialty care | | |
| 1. **** Tracking whether referrals to specialty mental health clinics are completed | | |
| 1. **** Contacting PC patients following missed referral appointments | | |
| 1. **** Working with PC staff to make appropriate treatment decisions | | |
| 1. **** Working with MH specialty staff to make appropriate treatment decisions | | |
| 1. **** Facilitating clinic adherence to guidelines | | |
| 1. **** Watchful waiting and monitoring of subsyndromal depression | | |
| 1. **** Watchful waiting and monitoring of individuals who initially resist engagement in treatment | | |
| 1. **** William Miller’s methods for Motivational Interviewing | | |
| 1. **** Problem Solving Treatment | | |
| 1. **** Cognitive Behavior Therapy | | |
| 1. **** Group Therapy | | |
| 1. **** Mental health “curbside consultation” for PC providers from integrated care clinical staff | | |
| 1. **** Mental health “curbside consultation” for PC providers from mental health specialty clinical staff | | |
| 1. **** Diagnosing psychiatric illnesses | | |
| 1. **** Advising PC providers on prescribing psychiatric medications | | |
| 1. **** Prescribing psychiatric medication | | |
| 1. **** Crisis/emergency intervention (for example, suicide intervention) | | |
| 1. **** Championing/marketing the program to PC staff | | |
| 1. **** Championing/marketing the program to MH staff | | |
| 1. **** Fostering patient activation (in other words, helping patients to become informed and active in their own care) | | |
| 1. **** Providing patient education materials | | |
| 1. **** Education focused discussions with patients | | |
| 1. **** Any other integrated care activities?   **S4_28a** List:_________________________________ | | |
| 1. Does the integrated care program limit the amount of services provided before referring to specialty mental health services? | **YES** | **NO🡪Skip to S5_1** |
| 1. Does it limit the number of encounters? | **YES** | **NO 🡪Skip to S4_32** |
| 1. What is the maximum number of encounters? _____ |  |  |
| 1. Does it limit the number of weeks | **YES** | **NO🡪Skip to S4_34** |
| 1. What is the maximum number of weeks? ______ |  |  |
| 1. Does it limit program use by some other criteria? | **YES** | **NO🡪Skip to S5_1** |
| 1. List criteria:_________________________________ |  |  |
| 1. What is the time limit?_____________________ |  |  |

**Section 5: Referrals to the Integrated Care Program**

1. I am going to read you a list of professions. Please tell me if these individuals can refer to your clinic’s Integrated Care program.

| 1. **** Primary Care physicians |
| --- |
| 1. **** Physician Assistants or Advance Practice Nurses (for example, nurse practitioners) |
| 1. **** Primary Care RNs |
| 1. **** PC-based Medical Social Workers (i.e., not integrated care) |
| 1. *If have integrated psychiatrists as well as other integrated staff*:   **** PC-based integrated psychiatrists (in other words, co-located in PC) can refer to other integrated clinical staff members |
| 1. *If have integrated psychologists as well as other integrated staff*:   **** PC-based integrated psychologists (in other words, co-located in PC) can refer to other integrated clinical staff members |
| 1. *If have integrated social workers as well as other integrated staff*:   **** PC-based integrated social workers (in other words, co-located in PC) can refer to other integrated clinical staff members |
| 1. **** MH-based specialty care psychiatrist (in other words, not co-located in PC) |
| 1. **** MH-based specialty care psychologist (in other words, not co-located in PC) |
| 1. **** MH-based specialty care social worker (in other words, not co-located in PC) |
| 1. **** Patients can refer themselves |
| 1. **** Other: |
| 1. Please list: _____________________________________ |

1. Now, I am going to read you a list of possible ways that providers can refer patients to, or engage them in, the Integrated Care program. Please tell me if your clinic’s program uses this method.

| 1. **** Electronic medical record (CPRS) consults from PC provider |
| --- |
| 1. **** “Curbside consultations” with Integrated Care program staff |
| 1. **** Direct “hand-offs” of patients from PC providers to Integrated Care providers (for example, PC provider walks patient over to Integrated Care provider) |
| 1. **** Integrated Care staff call patient following a conversation with PC provider |
| 1. **** Care manager calls co-located Integrated Care provider |
| 1. **** PC providers suggest to patients that they self-refer |
| 1. **** Other: 2. Please list: ___________________________________________ |

1. Which of the following statements are true about your clinic’s Integrated Care program?

| 1. All positive depression screens are automatically referred to the clinic’s Integrated Care program |  True |  False |
| --- | --- | --- |
| 1. All positive PTSD screens are automatically referred to the clinic’s Integrated Care program |  True |  False |
| 1. All positive Alcohol screens are automatically referred to the clinic’s Integrated Care program |  True |  False |

**Section 6: Assessment with Structured Tools and Laboratory Tests**

| 1. Do Integrated Care staff conduct **initial** mental health assessments with structured tools and/or laboratory tests? | **YES** | **NO** | **If NO to both, skip to Section 7** |
| --- | --- | --- | --- |
| 1. Do Integrated Care staff conduct **follow-up** mental health assessments with structured tools and/or laboratory tests? | **YES** | **NO** |  |
| **If YES to S6_1 but NO to S6_2, skip to item S6_4.** | | | |
| 1. How often does Integrated Care staff conduct follow-up assessments? **[can only select one]** | | | |
| **** As determined by Integrated Care program protocol **OR** | | | |
| **** As determined by patient need and clinical judgment without the use of a protocol **OR** | | | |
| **** Other   - 1. describe:______________________________________ | | | |

1. I am going to read a list of tests and tools. Please tell me which of these your clinic’s Integrated Care program uses:

| 1. **** Demographic tool |
| --- |
| 1. **** Depression assessment tool (separate from mandated screening) |
| Does your clinic’s program use any of the following depression assessment tools?   - 1. **** PHQ-9 |
| - 1. **** Depression assessment tools other than PHQ-9 (for example, Beck Depression Inventory, Hamilton)? |
| 1. **** Past and current depression treatment history |
| 1. **** Laboratory tests for positive depression screen (for example, CBC, BMP, TSH, B-12) |
| 1. **** Anxiety assessment tool |
| Does your clinic’s program use any of the following anxiety tools?   1. **** Full anxiety assessment tool (for example, STAI, BAI, GAD-7, MINI Module for Generalized Anxiety Disorder)   *Provide full names if asked:*  STAI = State-Trait Anxiety Inventory  BAI = Beck Anxiety Inventory  GAD-7 = General Anxiety Disorder-7 question  MINI = Mini-International Neuropsychiatric Interview |
| 1. **** Short anxiety screen (for example, TIDES screener) |
| 1. **** Panic disorder assessment tool |
| Does your clinic’s program use any of the following panic disorder assessment tools?   1. **** Full panic disorder assessment tool (for example, MINI Module) |
| 1. **** Short panic disorder screener (for example, TIDES screener) |
| 1. **** PTSD assessment tool |
| Does your clinic’s program use any of the following PTSD assessment tools?   1. **** Full PTSD checklist |
| 1. **** Short PTSD screen (for example, TIDES screener) |
| 1. **** Audit-C for alcohol misuse or abuse |
| 1. **** Alcohol and/or illicit drug use history |
| 1. **** Mania assessment tool |
| 1. **** Psychosis assessment tool |
| 1. **** Suicide ideation assessment tool |
| 1. **** Functional status assessment tool (for example,SF-12) |
| 1. **** Blessed Orientation-Memory-Concentration Test or similar cognitive screening measure for patients age 50 and over or with suspected cognitive impairment |

**Section 7: Program Monitoring, Evaluation, and Quality Improvement**

| 1. Does your facility conduct any Integrated Care program monitoring, evaluation or quality improvement activities? | **YES** | **NO🡪Skip to S8_1** |
| --- | --- | --- |
| 1. I am going to read you a list of program monitoring and quality improvement activities. Please tell me whether your facility conducts each of these. | | |
| - 1. **Ask only if YES to depression assessment tools (S6_4b, S6_4b1, or S6_4b2).**   **** Monitoring outcomes, (for example, monitoring PHQ-9 or other depression assessment tool scores over time) | | |
| - 1. **Ask only if YES to care management (S3_1).**   **** Assessing effectiveness of care manager-provider communication (for example, appropriately acknowledging and responding to each other; ensuring that contact lists are up-to-date) | | |
| - 1. **** Seeking out provider feedback about Integrated Care program and making appropriate changes | | |
| - 1. **** Seeking out patient feedback about the Integrated Care program and making appropriate changes | | |
| - 1. **** Assessing integrated care program performance (for example, number and appropriateness of referrals) | | |
| - 1. **** Monitoring patient wait times to see Integrated Care clinical staff and making necessary staffing adjustments | | |

**Section 8: Electronic tools**

1. I am going to read you a list of electronic tools. Please let me know which of these, if any, your clinic uses for primary care mental health integration.

| 1. **** View alerts |
| --- |
| 1. **** Touch pads, kiosks, or other electronic media for administering structured assessment instruments |
| 1. **** Depression case finder (for example, IT patch) to search for new PC anti-depressant prescriptions |
| 1. **** CPRS co-signs (in other words, PC providers co-sign Integrated Care program staff members notes) |
| 1. **** Automated tracking |
| 1. **** Behavioral Health Lab (BHL) integrated care software package |
| 1. **** TIDES integrated care software package |
| 1. **** Other electronic tools |
| **S8_1h1** Please describe: ___________________ |

**If not using any tools or if only using ‘View Alerts (S8_1a) and/or ‘CPRS Co-signs’ (S8_1d), skip to section 9.**

| 1. Does your clinic’s local IT staff maintain your electronic tools? | **YES** | **NO** |
| --- | --- | --- |

**Section 9: Suicide prevention protocol**

| 1. Does this facility review, test, and monitor its suicide prevention protocol? | **YES** | **NO🡪 skip to S10** |
| --- | --- | --- |
| 1. How often does this monitoring occur? |  On regular basis |  On an as needed basis |

**Section 10: Communication Between Services**

1. Please rate your answer to the following question on a scale of 1 to 7 where 1 is not at all, 4 is a moderate amount, and 7 is a great deal: To what extent do your **clinic** **level** PC and MH clinical leaders engage in activities that promote effective two-way communication between PC and MH services?

| **Not at all** |  | **A moderate amount** | | |  | **A great deal** |
| --- | --- | --- | --- | --- | --- | --- |
| 1 | 2 | 3 | 4 | 5 | 6 | 7 |
|  |  |  |  |  |  |  |

1. Using the same scale: To what extent do the **VAMC level** directors of PC and MH engage in activities that promote effective two-way communication between PC and MH services?

| **Not at all** |  | **A moderate amount** | | |  | **A great deal** |
| --- | --- | --- | --- | --- | --- | --- |
| 1 | 2 | 3 | 4 | 5 | 6 | 7 |
|  |  |  |  |  |  |  |

**Section 11: Models and Training**

| 1. Did the primary care staff members attend an orientation or initial training meeting? | **YES** | **NO** |
| --- | --- | --- |
| 1. Did the mental health staff members attend an orientation or initial training meeting? | **YES** | **NO** |
| 1. Have any of your clinic’s integrated care staff members attended a formal training offered by care management experts? | **YES** | **NO** |
| 1. *Ask if have care manager(s):* Has your clinic’s care manager (or have any of your clinic’s care managers) attended such a training? | **YES** | **NO** |
| 1. Has your clinic used the TIDES training manuals for your integrated care program staff? | **YES** | **NO** |
| 1. Have any of the integrated care staff attended a TIDES training seminar? | **YES** | **NO** |
| 1. Do any of the integrated care staff use TIDES VistA and CPRS tools and templates? | **YES** | **NO** |
| 1. Have any of the integrated care staff gone through a formal TIDES care management training? | **YES** | **NO** |
| 1. Have the integrated care staff used the BHL Core Assessment? | **YES** | **NO** |
| 1. Have any of the integrated care staff attended a BHL training seminar? | **YES** | **NO** |
| 1. Have the integrated care staff used BHL training manuals? | **YES** | **NO** |
| 1. Have any of the integrated care staff participated in the biweekly BHL national phone calls with the model developers from the Philadelphia VA? | **YES** | **NO** |
| 1. Have any of the integrated care staff ever consulted with the BHL model developers? | **YES** | **NO** |
| 1. Does your clinic’s integrated care program incorporate the TIDES depression care manager model? | **YES** | **NO** |
| 1. Does the integrated care program incorporate the Behavioral Health Lab (BHL) model? | **YES** | **NO** |
| 1. Does the integrated care program include co-located collaborative care (e.g., White River Junction)? | **YES** | **NO** |

**Section 12: Closing**

That is all the questions we have about your integrated care program. Do you have any additional comments that you would like to share regarding your program or PC-MH integration generally?

As we mentioned, we may need to speak with you again in the future. We will let you know in advance that we would like to speak with you again.

**Section 13: Respondents**

| **Position** | **Profession** | **ID #** |  |
| --- | --- | --- | --- |
| Integrated care provider |  |  |  |
| Primary care clinic leader |  |  |  |
|  Primary care program champion (non-clinic leader) |  |  |  |
| Other primary care provider |  |  |  |
| Mental health clinic leader |  |  |  |
|  Mental health program champion (non-clinic leader) |  |  |  |
| Other mental health provider |  |  |  |
| Other1; describe: |  |  |  |
| Other2; describe: |  |  |  |
| Other3; describe: |  |  |  |
| Other4; describe: |  |  |  |
| Other5; describe: |  |  |  |
| Other6; describe: |  |  |  |
| Other7; describe: |  |  | |
| Other8; describe: |  |  | |
| Other9; describe: |  |  | |

**Section 14: Interviewer’s Comments**
