# Supplementary material for: Using implementation facilitation to foster clinical practice quality and adherence to evidence in challenged settings: a qualitative study
Source: BMC Health Serv Res. 2017 Apr 20;17:294. doi: 10.1186/s12913-017-2217-0 (PMC5397744; doi:10.1186/s12913-017-2217-0)
Supplement: Supplementary file 2 — PC-MHI program components summary template. Template for creating a summary of the components of each facility’s program for integrating mental health services into primary care. Designed for use with the “PC-MHI Program Component Assessment Interview Guide.” (DOCX 52 kb) [file 12913_2017_2217_MOESM2_ESM.docx]

**Primary Care-Mental Health Integration (PC-MHI)**

**Program Components Summary Template**

Purpose

This template was designed to summarize responses recorded on the *PC-MHI Program Component Assessment Interview Guide***.**

Rater ID: _____________ Program ID: ___________

Note: NA = Not applicable; DNK = Do not know

1. **Size of clinic at start of study**
2. **Availability of MH specialty care at start of study**

*[Use categories: full on-site clinic, limited on-site with therapists or prescribers, none, DNK]*

1. **PC-MHI Program staff**

| **Staff Type** | **% effort** | **Location** | | **PC-MHI Role** | | |
| --- | --- | --- | --- | --- | --- | --- |
|  |  | **Co-located** | **Central** | **Prescriber** | **Therapist** | **Care Manager** |
|  |  |  |  |  |  |  |
|  |  |  |  |  |  |  |
|  |  |  |  |  |  |  |

[*Use * in table and enter notes here such as: *Serves multiple clinics and cannot determine exact percentage of time for this clinic]*

1. **Conditions PC-MHI addresses**
2. **PC-MHI includes Co-located Collaborative care? YES**  **NO**

***Co-located Care Characteristics***

| **Provider type** | **Immediate**  **access** | **Average wait** | **Average length** | **Available all clinic hours** | **APN access to psychiatrist** |
| --- | --- | --- | --- | --- | --- |
| Prescriber(s) |  |  |  |  |  |
| Therapist(s) |  |  |  |  |  |

***Extent of co-located and PCPs’ communication*** (1=not at all; 7=a great deal):

- - To incorporate patient preferences into treatment plans: ____
  - To develop and review treatment plans: ____

1. **PC-MHI includes care management? YES**  **NO**

***Care management model***:  BHL  TIDES  Unknown

| ***Care Management Characteristics*** | | **Yes** | **No** |
| --- | --- | --- | --- |
|  | Care manager typically contacts patients by phone rather than face-to-face |  |  |
|  | PCPs always remain involved |  |  |
|  | Care manager has access to CPRS MH Assistant |  |  |
|  | For suicidal patients, care manager has way to contact appropriate personnel |  |  |
|  | For suicidal patients, on call MH provider trained in suicide prevention is available |  |  |

***Conditions care manager addresses***: *[list, not bullets]*

***% of PC staff that have met with care manager***: _______

***Supervision frequency***: _____________________________

***Supervision provided by***: ____________________________

***Extent of care managers’ and PCPs’ communication*** (1=not at all; 7=a great deal):

- - To incorporate patient preferences into treatment plans: ____
  - To develop and review treatment plans: ____

1. **Model-specific material/resource use**

| **Model** | **PC-MHI**  **Training** | **Use manuals** | **Use tools/ templates** | **BHL phone calls** | **Consulted w/ BHL model developers** |
| --- | --- | --- | --- | --- | --- |
| BHL |  |  |  |  |  |
| TIDES |  |  |  |  |  |

1. **Other PC-MHI Program Characteristics**

***Service limits before referral to specialty MH care***: ______________

***Referral to program***:

***Referral sources***: __________________________________

***Referral methods***: __________________________________

***Screens that are automatically referred***: ________________

***Program monitoring, evaluation, and QI***: _______________________

***Electronic tools***: _______________________ ***IT staff maintain***: **YES**  **NO**

***Staff attended orientation/initial training meetings***  PC staff  MH staff

1. **Facility characteristics**

***Frequency facility monitors its suicide prevention protocol: __________***

***Extent that PC and MH leaders engage in activities promoting effective 2-way communication*** ***between their services*** (1=not at all; 7=a great deal):

- - At the clinic level: ____
  - At the VAMC level: ____

1. **Assessment with structured tools and lab tests:** *[If none, type none here and delete other items]*

At initial assessment  At follow up assessment

***Follow-up Assessment Frequency***: _______________________

***Tools and tests***: _______________________________________

1. **PC-MHI Services** *[all on one page]*

Mental health assessment using formal tools such as the PHQ-9

Ongoing monitoring of patient progress

Clinical psychiatric evaluation without formal assessment tools

Facilitating or providing advice about referrals to mental health specialty care

Direct referral and/or transfer to mental health specialty care

Tracking whether referrals to specialty mental health clinics are completed

Contacting PC patients following missed referral appointments

Working with PC staff to make appropriate treatment decisions

Working with MH specialty staff to make appropriate treatment decisions

Facilitating clinic adherence to guidelines

Watchful waiting and monitoring of subsyndromal depression

Watchful waiting and monitoring of individuals who initially resist engagement in treatment

William Miller’s methods for Motivational Interviewing

Problem Solving Treatment

Cognitive Behavior Therapy

Group Therapy

Mental health “curbside consultation” for PC providers from integrated care clinical staff

Mental health “curbside consultation” for PC providers from mental health specialty clinical staff

Diagnosing psychiatric illnesses

Advising PC providers on prescribing psychiatric medications

Prescribing psychiatric medication

Crisis/emergency intervention

Championing/marketing the program to PC staff

Championing/marketing the program to MH staff

Fostering patient activation

Providing patient education materials

Education focused discussions with patients

Other integrated care services/activities: ___________________________________

| Template Authors:  Mona J. Ritchie, PhD, MSW and Louise E. Parker, Ph.D.  For information about this instrument and how to utilize it, please contact:  Mona J. Ritchie, PhD, MSW  Implementation Coordinator  VA QUERI Program for Team-Based Behavioral Health  Phone: (501) 257-1735  Email: [Mona.Ritchie@va.gov](mailto:Mona.Ritchie@va.gov) |
| --- |
